# The Gut Microbiome and the Big Eight

**DOI:** 10.3390/nu12123728

**Published:** 2020-12-03

**Authors:** Cassandra Suther, Matthew D. Moore, Avraham Beigelman, Yanjiao Zhou

**Affiliations:** 1 Department of Food Science, University of Massachusetts, Amherst, MA 01003, USA; csuther@umass.edu (C.S.); mdmoore@umass.edu (M.D.M.); 2 Department of Medicine, University of Connecticut Health Center, Farmington, CT 06030, USA; 3 Kipper Institute of Allergy and Immunology, Schneider Children’s Medical Center, Tel Aviv University, Tel Aviv 5891000, Israel; beigelmana@wustl.edu

**Keywords:** food allergy, microbiome, dysbiosis, short-chain fatty acids, cow milk allergy

## Abstract

Food allergies are increasing at an alarming rate, with 6.5% of the general population affected. It has been hypothesized that the increase in allergies stems from the “hygiene hypothesis”. The gut microbiome, a collection of microbiota and their genetic contents from the gastrointestinal tract, has been shown to play a part in the development of food allergies. The Food and Drug Administration requires all regulated food companies to clearly state an inclusion of the major, or “big eight” food allergens on packaging. This review is to provide information on the significant advancements related to the gut microbiome and each of the eight major food allergies individually. Establishment of causal connection between the microbiome and food allergies has uncovered novel mechanisms. New strategies are discussed to prevent future sensitization and reaction through novel treatments involving functional additives and dietary changes that target the microbiome.

## 1. Introduction

A food allergy is defined as an abnormal immune reaction to the repeated exposure of certain foods [1]. It can manifest as minor gastrointestinal distress and skin rashes, to life-threatening anaphylactic shock [1]. These adverse symptoms and the burdens associated with avoiding food products can disrupt quality of life. It is unknown why some individuals will develop an allergy to a specific antigen, while others will not [2].

Food allergies have been rising at an alarming rate, nearing 6.5% (5% of adults and 8% of children) of the general population (of developed counties) affected to date [1]. Of these food allergies, 90% are caused by “The Big Eight”, a term referring to all major Food and Drug Administration (FDA)-regulated food products. These foods include cow milk, hen’s egg, fish, crustacean shellfish, tree nut, peanut, wheat, and soybean [1]. It has been hypothesized that the increase in allergies stems from the “hygiene hypothesis”, which states that early life exposure to microorganisms protect against allergic disease [1]. The gut microbiome, termed a collection of microbiota and their genetic contents in the gastrointestinal tract, has been shown to play a part in the development of asthma, atopic dermatitis, and food allergies through mucosal tolerance and possible bacterial metabolites over the past decade. 

**Figure 1:**
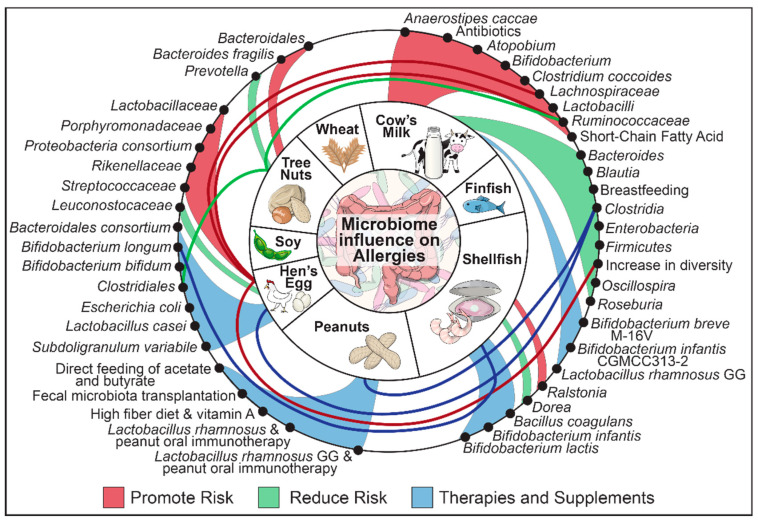
Summary of bacterial and dietary changes involved in food allergy.

Despite being a very timely and important topic, no recent reviews exist addressing the gut microbiome and its relationship with allergens specific to each of The Big Eight foods. This review will present the gut microbiological and dietetic factors associated with the development and treatment of food allergies. Food antigens will be explored individually for their connection to the gut microbiome (Figure 1).

## 2. We Are What We Eat: Diet and the Microbiome

Trillions of gut microbes from thousands of different species make up the highest density of microbes within the human body [3]. Diet shapes the configuration of the gut microbiome at early life. The gut microbiome of infants that were breastfed have a unique and beneficial composition that is not observed in those given formula when compared later in life [4]. Gut microbiome dysbiosis in early life is thought to be related to development of allergies later in life. When children start a solid food diet (at approximately 6 months), the gut microbiome shifts significantly for a second time. The adult gut microbiome generally can be classified into three distinct enterotypes that are dominated by *Bacteroides, Prevotella*, or *Ruminococcus,* respectively [4]. The diet of the individual influences the enterotypes, with *Bacteroides* being associated with a Western-type diet high in proteins and fat, and *Prevotella* being associated with plant fiber consumption. As a modifiable target, modulation of the gut microbiome through dietary intervention (high fiber-related diet), prebiotics or probiotics have seen an increase in both research interest and product development in recent years. However, the magnitude and duration of microbiome changes by dietary intervention have been largely inconsistent.

High levels of fiber can increase the production of short-chain fatty acids (SCFAs) from specific bacteria in Firmicutes, such as Bacteroidetes, Clostridia (*Ruminococcus* enterotype) and Bacilli [5]. SCFAs are bacterial fermentation products and are profoundly affected by food intake. There are three common short-chain fatty acids that tend to be produced by bacteria: acetate, propionate, and butyrate. Acetate is the acid produced in the highest quantity, but butyrate is thought to be the main energy source for colonocytes. These acids are absorbed into the portal vein during lipid digestion, and have been associated with major health benefits, including a reduced risk of inflammatory diseases and Treg function [6,7]. A decrease in SCFAs may lead to an increase in pathogenic bacteria due to a decrease in gut pH [8]. Pathogenic bacteria can cause epithelial damage to the colon walls, and many believe food allergies are an epithelial barrier disease [9].

Other metabolites derived from commensal bacteria include long-chain fatty acids (LCFAs), glycolipid, histamines, vitamin B2/B9 byproducts and amino acids [10,11]. LCFAs are major nutrients, including the clinically important ω3 and ω6 FAs, with ω3 FAs known to have anti-allergic and anti-inflammatory properties [10]. Pro-inflammatory LCFA metabolites, including stigma- and sitosterols and 8-hydoxyoctanoate, are associated with lower risk of allergy development [10]. These metabolites are thought to decrease IL-4 produced Th2 cells. 

The current dietary interventions for those with food allergies include tolerance or simply avoidance. The enrichment of SCFA-producing bacteria in the body has been investigated as a potential treatment, as stated above, using the enrichment of the Firmicutes phylum. One may naturally increase these bacteria by introducing higher levels of fiber, or prebiotics, into their diet. High-fiber diets used in mice were found to decrease allergic sensitization [12]. Other options for dietary changes may include an increase in food-derived probiotic bacteria found within fermented foods. Histamine can be derived from bacterial and dietary sources, including fermented foods. It should be noted that dietary and bacterial-derived histamine’s role in food allergy is still currently unknown. However, some data suggest that the intestinal inflammation brought on from dietary histamine may enhance sensitization [11]. Western lifestyle and diet promote a change in bacteria which has not been associated with the human gut in evolution. These changes from a millennia of human biology, even small, may lead to larger detrimental outcomes. 

## 3. Just One Bite, or Maybe Two: The Mechanisms of Food Allergy

Certain food may cause non-immune responses in the body, including digestive enzyme disorders. However, these should not be confused with true food allergies. Food allergies are atopic disorders in which the body has a hypersensitive immune response to a normally harmless food-derived protein [2]. 

The most common cause of food allergy is defined as an immediate IgE-mediated reaction. For an allergic reaction to occur, the antigen must interact with the intestinal mucosa multiple times. One will not have an allergic reaction to a food the first time they ingest it. This process is defined as sensitization. For food sensitization to occur, the protein must get to the intestinal epithelium without denaturing. They are then transferred from the lumen into the mucosa through gut epithelial cells and other specialized cells [13]. Once in the mucosa of the gut, mucosal dendritic cells interact with the antigen and proceed to T cell locations to present the antigen to naive T cells, to form the classically seen Th2 cells. Th2 cells will produce certain cytokines, including IL-3, IL-4, IL- 5, IL-9, and IL-13. From this, B cells will proliferate to produce IgEs, in place of other antibodies like IgG or IgM. These IgEs have a high affinity for FcεRI receptors found on mast cells and basophils and gear the cells up for further exposure, as sensitization is now complete [2]. The processes of sensitization can occur in several other locations in the human body, including the oral cavity, skin, and respiratory tract [13]. 

When there is reintroduction of the antigen, anaphylactic degranulation of the mast cells and basophils follows and there is a release of inflammatory mediators, including histamine, cytokines, and leukotrienes. At this point, the presence of the food antigen in any body tissue (mouth, stomach, gut) can induce an IgE-mediated reaction that generally occurs a few minutes after exposure. Those with IgE-mediated or mixed food allergies can be identified based on the detection of food allergen-specific IgE. 

Other forms of food allergy include delayed non-IgE-mediated reactions and pollen food syndrome (PFS), which are less common and often confused for IgE-mediated food allergies [2,14]. Delayed non-IgE-mediated food allergies involve antigen contact with sensitized T lymphocytes, as opposed to mast cells, which then damage the gut mucosa. This type of reaction is associated with disorders such as celiac disease and may occur hours later, involving vomiting and diarrhea [2]. Interestingly, pollen food syndrome is still an (IgE)-mediated disease. However, it is a consequence of an already established pollen allergy cross reacting with antigens, similarly structured to pollen, found within certain foods [14]. This is commonly seen in nuts, fruits, and vegetables. However, both are not within the scope of this review.

## 4. “Good Source of Protein?”: Interactions of Food Matrices and Gut Permeability

Allergy type and dosage can affect immune response, along with adjuvants in the gut. The more food protein that makes it intact to the gut, the more chance there is of sensitization. While most are broken down by gastric acid and digestive enzymes, some intact proteins and peptides can move further into the large intestine. Certain factors can affect sensitization by allowing for a strong or weaker epithelial wall; including genetics, alcohol, anti-inflammatory drugs, pathogens, and stress [2]. Gut bacterial populations and bacteria-derived proteases and peptidases may affect protein absorption into the blood stream [15]. Cooking promotes protein denaturation through various forms of heat processing (pasteurization, blanching, convection/conduction). However, peanuts and tree nuts are commonly roasted and many of their allergic proteins are not broken down in the process [16]. It is also believed that the heating of peanuts and shrimp may enhance allergenicity, by aggregating protein to be more resistant during digestion [17]. 

Along with this, a few of The Big Eight food allergens can be consumed uncooked in some cases. Soybeans, finfish, and shellfish are commonly eaten raw. These uncooked or harder-to digest-factors, along with a higher protein content, may contribute to why these foods are considered to cause more sensation in individuals than other foods. Interestingly, many children may tolerate baked milk and egg if given in a wheat matrix, as other ingredients eaten together are known to decrease the proteins availability to interact with the epithelial wall [17].

It is currently unclear why these food proteins cause a negative immune response in some. The gut-associated lymphoid tissues maintain tolerance by differentiating against self or non-self-antigens and through the recognition the pathogens [18]. However, if this process breaks down, the body can no longer tell the different between friend or foe. Tolerance can be achieved to negate harmful effects. Oral tolerance to a food allergen can develop after frequent expose to the antigen due to possible changes and mechanisms involved in the dendritic cells, gut epithelial cells, and the gut microbiome. However, at this time, the exact mechanisms of desensitization is unknown [13].

## 5. The Gut, The Big Eight and the Correlation between the Two

These is a clear difference between each food allergy, both in sensitization period and severity of reaction. Children tend to outgrow cow milk and hen’s egg allergies, but peanut and shellfish allergies can occur later in life and may be very severe. Table 1 displays the different food proteins that have been found to induce allergic reactions.

### 5.1. Cow Milk

Often confused with lactose intolerance, a cow milk allergy (CMA) can be a reaction to the main two proteins in milk, casein, and whey (Table 1). This allergy affects 2–6% of children, making it the most common childhood food allergy [27]. The typical onset age for milk allergy is 4.3 months into life, or when exposed to milk after breast feeding [28]. Most children become tolerant by the age of three, but some do not show resolution until teenage years [29]. Milk allergies can leave children with a challenge of getting proper nutrition. Infant formula typically comprises a blend of cow milk whey and casein, vegetable fat, lactose sources, vitamin/mineral mix, and probiotic bacteria [30]. Because of the use of cow milk protein in these typical formulations, a substitute is required. Formula is used widely, as only 38% of newborns exclusively breastfed globally. In the United States, only 75% of infants are exclusively breastfed starting from birth, with 67% of all children relying on infant formula for some portion of their nutrition after three months. Only 13% of new mothers meet the recommendation of breastfeeding exclusively for six months [30]. Exclusive breastfeeding has been linked to a reduction in specifically cow milk sensitization. However, it should be noted that many different studies have been carried out, with conflicting results between breastfeeding, formula feeding, and milk allergy [31,32]. 

The exact mechanism as to why certain children will develop CMA remains poorly understood [33]. Increasing evidence suggests that a normal gut microbiome is critical to suppress CMA. Studies have shown germ-free animals to be at a higher risk for sensitization to cow milk protein [34,35,36,37]. Maternal and infant use of antibiotics has been associated with an increased risk of the child developing a CMA [38]. In addition, compositions of the early life gut microbiome differ between CMA resolution and continuing allergy in children, with enrichment of Clostridia and Firmicutes in the infant gut microbiome of those whose CMA resolved [39]. In a study of 226 children, little over half (56%) had the allergy resolved, with the gut microbiome composition between the two groups being significantly different between 3 and 6 months. This was not seen between other ages, supporting the importance of the early life gut microbiome in CMA resolution. 

There are two different theories involving the Clostridia class from Firmicute phylum and its involvement in milk allergy. One theory states Firmicutes and those in the Clostridia class are beneficial, but others claim they may be harmful. Bacteria from Clostridia are major butyrate producers. Butyrate is known to regulate colonic regulatory T cells that are essential for immune tolerance [40,41]. Butyrate also allows for regulation of the intestinal epithelial barrier which may decrease the intake of food antigens into the blood. Lower abundance of the Firmicutes phylum and the Clostridia class has been seen in children whose allergy did not resolve [39]. However, it is theorized that not all Firmicutes and products of the Clostridiales species lead to protection, but only specific species and even strains. In one study, treatment with extensively hydrolyzed casein formula combined with *Lactobacillus rhamnosus* GG leads to an increase in Firmicutes, including *Roseburia, Blautia* and *Coprococcus* (all from the Clostridiales order), in both allergic and healthy groups [42]. *Oscillospira* was only seen in those whose allergies resolved. In their analysis of fecal butyrate levels, higher levels were found in those who became tolerant to milk allergies. Interestingly, there were strain-specific differences of *Blautia* and *Roseburia* between the tolerant and allergic patients. It should be noted that this study was conducted on stool after allergic symptoms start to appear, and that the importance of an increased abundance of Firmicutes may be during sensitization. The gut microbiome develops in early life and becomes mature at 2–3 years old [4]. Longitudinal characterization of the microbial dynamics before and after sensitization and challenging will provide a holistic view of the evolving microbiome and development of CMA. Another Clostridia species, *Anaerostipes caccae*, has been shown to reduce the risk of developing CMA [43]. When using a fecal microbiota transplantation (FMT) from CMA children into mice, the mice became more susceptible to anaphylactic reactions to milk protein when compared to mice treated with healthy children’s stool, in which higher levels of Clostridiales and *Anaerostipes caccae* were found. Gene expression analysis of intestinal epithelial cells of recipient mice identified genes involved in pyruvate metabolism, *acot12* and *me1*, were upregulated in the ileum of CMA-colonized mice, compared to the healthy-colonized mice. An integrated analysis identified a strong negative correlation of *Anaerostipes caccae* with pyruvate metabolism. The latter was speculated to be one key intermediate during glycolysis, a metabolic pathway for colonocytes during gut microbiome dysbiosis. This study highlights that host-microbiome interaction is central to regulating tolerance to dietary antigens.

The counterargument for the beneficial effects suggests that the higher concentration of butyric acid produced by bacteria in the Firmicutes phylum can increase the permeability of intestinal mucosa, thus possibly allowing higher amounts of cow milk protein to enter the bloodstream [39,44]. A metagenome functional prediction conducted on the stool of allergic children found a decrease in fatty acid metabolism in children whose allergy resolved [39]. It was suggested that cow milk lipids drive proinflammatory effects, and a decrease in fatty acid metabolism may be positively correlated with less inflammation. Other studies have found lower levels of branched short-chain fatty acids in healthy infants compared to allergic infants [45]. This study also found high levels of *Clostridium coccoides* in those with a cow milk allergy, along with an increase in butyric acid. Other studies have also found increased levels of *Clostridium coccoides* in groups which did not develop tolerance to CMA [46]. However, as stated above, it is important to remember that it is possible not all Firmicutes may lead to protection, even being as specific as species and strains. 

Introduction of bacteria that are suppressive of CMA has long been proposed to both preventing sensitization or aiding immunotherapy. Lower counts of probiotic bacteria, *Bifidobacteria* and *Lactobacilli*, have been reported in the guts of children with CMA [28]. The clinical outcome by reintroduction of these bacteria is mixed. *Lactobacillus casei* CRL431 and *Bifidobacterium lactis* Bb-12 were given to infants diagnosed with CMA for 12 months [29]. However, it was found that these bacteria did not have any effect on the acceleration of milk allergy tolerance. Comparatively, a similar study was conducted, which looked at the most common probiotic on the market, *Lactobacillus rhamnosus* GG and its ability to accelerates tolerance acquisition in infants with cow milk allergy [47], as *L. rhamnosus* GG has been shown to promote respiratory and gut immunity [48,49]. After 6 months of *Lactobacillus rhamnosus* GG supplementation, 60% of the infants with CMA had resolved symptoms, compared to the 22% in the control group. It was hypothesized that the resolution of CMA could be related to the immunoregulatory role of the *Lactobacillus rhamnosus* GG. *Lactobacillus rhamnosus* GG is found to balance generation of Th2-related cytokines [47]. With a more in-depth analysis of *Lactobacillus rhamnosus GG*, the probiotic was found to increase specific butyric acid-producing bacteria in the gut of children whose allergies resolved after treatment [42]. 

*Bifidobacterium* is another common probiotic used outside of the *Lactobacillaceae* family and is commonly found in the gut of infants. An increase in these bacteria has been seen to improve the characteristics in other IgE or Th2 allergies [50,51]. *Bifidobacterium breve* M-16V and a supplement containing a mixture of short-chain galacto-oligosaccharides and long-chain fructo-oligosaccharides, was introduced into the mice’s diet and the anaphylactic reaction to milk protein was measured [52]. A combination of both was found to reduce ear swelling upon cow milk protein introduction in the mice, with less effectiveness when used separately. The protective effect of *Bifidobacterium* in the mouse model of food allergy is also evident in another study where serum IgE levels were lower after administration of *B. infantis* CGMCC313-2, compared to that in controls [53]. A well-characterized healthy infant microbiota, containing high levels of *Bifidobacterium* and *Bacteroides,* was transplanted it into germ-free mice, which lead to an improved protection of milk allergy [36].

Unpasteurized milk is thought to protect from asthma and food allergy. However, its benefits are controversial [54]. Unpasteurized milk is known to contain many bacteria, including probiotics, which are otherwise inactivated in pasteurized milk. This includes lactic acid bacteria such, as *Lactobacillus*, *Streptococcus*, *Enterococcus*, *Lactococcus*, *Leuconostoc*, *Weisella* and *Pediococcus* [55]. However, possible contamination with numerous pathogenic bacteria, including *Staphylococcus aureus*, *Salmonella enterica*, and *Escherichia coli*, may lead to further gut damage [56]. Individuals should look for safer sources of probiotics, including other dairy products such as yogurts and cheese.

### 5.2. Hen’s Egg

Egg allergies affect 0.5% to 2.5% of young children, starting around infancy. However, this is as high as 8.9% of infants in certain populations [20], making it the second most common food allergen in children. In addition to causing dietary restrictions, egg allergies have significant health consequences for children, as they cannot receive certain vaccines generated using eggs. Ovomucoid is considered the main allergen in egg because of its immunoreactivity and heat resistance (Table 1). While ovalbumin (OVA) is the most abundant potential allergen in eggs, it is easily denatured during thermal processing [20]. Although most proteins are found within the egg whites, allergic children should avoid eggs altogether, as proteins can bleed into the yolk, along with the yolk having small amounts of allergic proteins themselves [57]. Tolerance may be accelerated similarly to milk, with studies finding some children able to tolerate eggs faster after a diet including baked eggs [58]. 

Early life gut microbiome composition of those with egg allergies has been shown to play an important role in development of the disease [59]. However, like the aforementioned milk studies, no association has been found between the gut microbiota and egg allergy resolution later in life (by the age of 8 years). Higher microbial diversity during this early stage of life has been found to correlate with egg allergy, sharing similar results to previous studies on other allergic diseases [59]. At the phylum level, Firmicutes and Verrucomicrobia are higher in infants with egg allergy, with *Ruminococcus* and *Lactococcus* associated on a genus level, when compared to health patients [59]. A difference in purine metabolism was also observed, with less in those with egg allergy. A depletion of uric acid inhibits the activity of xanthine oxidase in purine metabolism, significantly changing the inflammatory responses of mice. This has been seen in asthma exacerbation, a mechanism proposed to be involved in sensitization [60].

Bacterial interventions are a common research topic to alleviate egg allergy. Oral administration of *Bifidobacterium bifidum*, *Lactobacillus casei*, and *Escherichia coli* were found to lower IgE, or overall immune response in OVA with cholera toxin mice [61]; however, *E. coli* caused an unhealthy lack of weight gain. The differences in OVA-specific fecal IgA levels and OVA-specific serum IgG1 levels among the experimental bacteria suggest that they are likely to inhibit allergy responses through different mechanisms. *Bifidobacterium longum*, but not *Enterococcus faecalis,* extracellular vesicle–derived proteins were found to suppress egg allergies by induced apoptosis to bonded mast cells in mice [62]. 

The effect of other bacteria, that are not lactic acid bacteria, has also been investigated in the egg allergy mouse model to explore possible benefits [63]. Therapy with Clostridiales, *Subdoligranulum variabile* and a Bacteroidales consortium has been found to suppress allergy. In this study, a MyD88/ROR-γt pathway in nascent Treg cells was deficient in food allergy infants and mice. Inhibition of this pathway in Tregs abrogated protection. Oral supplements with 17 Clostridia strains, previously isolated and invested for positive effects, led to a reduction in OVA-specific IgE after sensitization and reduced diarrhea scores after challenge in mice [64].

OVA-sensitized mice with a mutation in the IL-4 receptor α chain (Il4raF709) harbor a distinct gut microbiome, with over-represented populations of *Lachnospiraceae, Lactobacillaceae, Rikenellaceae*, and *Porphyromonadaceae* [6]. Fecal transplantation of the microbiome in OVA-sensitized *Il4raF709* mice promoted OVA-specific IgE responses and anaphylaxis. Interestingly, treatment with OVA-specific Treg cells led to a suppression of allergic response, accompanied by the suppression of allergy associated microbes. Taken together, these reports suggest that specific gut microbiota and their associated immune effects are at least partially responsible for OVA-mediated food allergy, providing additional rationale for microbiome-based intervention in food allergy. 

### 5.3. Peanut

Peanut allergies are one of the most predominant food allergies to carry into adult life [65]. At least 11 peanut antigens have been described (Table 1). The quality of life for a child with a peanut allergy has been perceived to be worse than a diabetic child [66]. Peanuts can be easily hidden in foods, including their use as bulking agents in non-related items. Accidental exposure is common in children with peanut allergies, with annual incidence of 12–15% in these children [67]. There has been an increase in peanut allergies, with a 21% increase since 2010, with 2.5% of children in the U.S. having a peanut allergy [68]. Because of this and the severity of the reaction, peanut oral immunotherapy interventions have been developed [69]. More recently in 2020, the FDA has approved the first immunotherapy treatment, termed “*Palforzia*” (125,696) [70]. Oral immunotherapy can be described as an introduction of peanuts to children at a young age that show signs of peanut sensitization in hopes of increasing the threshold that induces a reaction. 

While *Palforzia* does not contain a bacterial component, many studies have included bacteria as an adjunct for the therapy. The first randomized placebo-controlled trial on the effectiveness of a combination of *Lactobacillus rhamnosus* GG and peanut oral immunotherapy lead to 89.7% of those who received treatment to be desensitized, compared to 7.1% in the no bacterial control group [71]. *Lactobacillus rhamnosus* GG was used in a different peanut oral immunotherapy, with 67% of the children in the treatment group and 4% in the placebo desensitized following the end of the trial [72]. It has been proposed *Lactobacillus* promotes peanut tolerance by enhancing the tolerogenic effects of cells, including regulation of T cells. Other treatments that have been investigated include FMT and a rationally defined bacterial consortium supplement. A Boston Children’s Hospital treatment in which children are given oral encapsulated frozen fecal microbiota transplantations (NCT02960074) [73] is set for completion in late 2020. Vedanta Biosciences has recently enrolled their first peanut allergy patient to receive VE416, an orally administered live biotherapeutic product containing a defined bacterial consortium. This is thought to have similar, if not more benefits than a FMT, without the need for inconsistencies in a live donor (NCT03936998) [74]. However, the potential impact of these new treatments remains to be seen. 

Dietary interventions have also been reported to have beneficial effects for peanut allergy. The introduction of a high-fiber diet, with vitamin A, in mice improved oral tolerance and protected against peanut allergy [12]. With only fiber and vitamin A, and not an added bacterial component, the gut microbial ecology of the mice was reshaped to have more diversity and an increase in Firmicutes, specifically Bacilli. In turn, this caused an increase in levels of acetate and butyrate. The direct feeding of acetate and butyrate in drinking water was also found to protect mice from peanut sensitization. This diet lead to an increase in tolerogenic CD103+ dendritic cells, which are required for oral tolerance to antigens, and higher IgA serum levels. Specifically, this IgA increase is thought to be associated with Clostridia. Mice treated with antibiotics or germ-free mice have been reported to be more susceptible to peanut sensitization [75]. Reintroduction of Clostridia bacteria, but not *Bacteroides* bacteria, blocks sensitization to peanut allergen in germ free mice [75]. The presence of Clostridia was thought to be associated with an adaptive expansion of Tregs found in the intestine and induce immunoglobulin class switching from IgE to IgA. It is believed that IgA contributes to the immune response by reducing allergen uptake [76]. Elevated levels of uric acid have been found in mice which have undergone sensitization to peanuts, as well as with children confirmed to have a peanut allergy [77]. Uric acid is a byproduct of purine metabolism, and the depletion of uric acid during sensitization of mice is known to prevent the development of IgE and IgG1. Changes in total antibody composition may help prevent sensitization. 

### 5.4. Tree Nuts

Tree nuts are a broad term used to describe any nut that grows on a tree, including almonds, brazil nuts, cashew nuts, hazelnuts, macadamia nuts, pecans, pistachios, and walnuts [22]. They differ from peanuts, which are classified as legumes along with peas and soybeans. Consumption of walnuts and other tree nuts has been seen to increase healthy probiotic and butyric acid-producing bacteria in the gut [78]. Approximately 4.9% of the general population has a tree nut allergy. The lack of this food source can have potential negative effects on health, including a lack of vegetable protein, dietary fiber, magnesium, potassium, copper, and vitamins E and K [22,79]. Peanut and tree nut allergies overlap within people commonly, as the structure between the antigenic proteins in both are similar. Unsurprisingly, a meta-analysis has found a stronger correlation between peanut and tree nut allergies [80]. Along with this, many nut allergies are due to an allergic reaction to pollen and not to the nut protein itself. This phenomenon is referenced to as pollen food allergy syndrome [81]. There are several different tree nut proteins that can cause allergy, most being found within the metabolic and storage protein family of the nut (Table 1) [81]. 

For those with tree nut allergies, a decrease in Clostridiales and higher Bacteroidales has been observed [80]. With peanuts and tree nuts taken together, a positive association can be seen in allergy with *Bacteroides*, *Bacteroides fragilis* and Bacteroidales, and a negative association with Clostridiales, *Prevotella* and *Ruminococcaceae* [80]. There is currently no microbiome associated treatments for tree nut allergies. However, like peanuts, there are serval immunotherapy options which have been studied [82,83]. As peanut and tree nut allergies are shown to have a similar structure between allergic proteins, positive results from bacterial tree nut immunotherapies may occur as it has with peanuts. Similarly, future work on utilizing certain bacteria as adjuncts to treatment for tree nut allergies should be investigated.

### 5.5. Crustacean and Molluscan Shellfish

Crustaceans are a subsection of the phylum Arthropoda and include crab, rock lobster, prawn, and shrimp. Mollusks generally consumed include gastropods (snail), bivalves (clam, oyster, scallop and mussel), and cephalopods (squid and octopus) [23], and belong to the phylum Mollusca. Tropomyosin is the predominant allergic protein in crustacean, while mollusks contain less well-understood allergens, along with different tropomyosin (Table 1). Mollusk allergies are known to cross react with other shellfish allergies regardless of these differences; however, crustacean and molluscan allergens do not cross react with fish allergens [23]. Recent studies have shown tropomyosins from house dust mites to also cause allergies in those allergic to shellfish trompmyosins [84]. However, vertebrate tropomyosins are not known to cause disease. As of 2019, crustacean and molluscan allergy is the most common food allergy affecting adults in the United States [85]. Importantly, while most of The Big Eight allergies develop in childhood, shellfish allergies can develop at any point in life, much like peanuts [86].

*Bifidobacterium* has been investigated to have a role in the protection of shellfish-related food allergy. *Bifidobacterium infantis* and *Bifidobacterium lactis*, bacteria commonly found in nursing mothers and the guts of infants, was found to reduce shellfish-specific IgE in mice [87,88]. *Bifidobacterium infantis* was found to revert bacterial proportion imbalance caused from the allergen, by increasing *Dorea* and decreasing *Ralstonia*. It was proposed that this proportion of *Dorea*/*Ralstonia* is involved in Treg cell differentiation and could help balance the Th2/Treg ratio. *Bifidobacterium lactis* was found to increase the ratio of Treg and Th17 cells in a mouse model. Other studies have found *Bifidobacterium longum* and *Bacillus coagulans* to regulate gut dysbiosis and mitigate overactive Th2 response in tropomyosin-induced allergic mice [89]. *Bifidobacterium longum* and *Bacillus coagulans* have also found to regulate gut arginine metabolism pathways, leading to the conclusion that metabolites of aspartate and arginine may be critical for prevention of food allergy. However, it should be noted that these studies mostly focused on *Bifidobacterium,* and the effects of other bacteria has not been as well investigated. Future work should focus on investigating the role of other commensal bacteria in mitigating shellfish allergies. 

### 5.6. Wheat

Gluten sensitivity is not a true wheat allergy. However, the insoluble gluten proteins gliadins and glutenins are reported to be the most responsible for inducing IgE-related sensitization, especially omega-5-gliadin (Table 1) [26]. Celiac disease is a common non-IgE-mediated reaction involving intolerance to gluten. The prevalence of wheat allergies varies between different regions of the world, ranging from 0.2% to 1% [26]. Like cow milk and egg allergies, wheat allergy is commonly outgrown by adulthood [65]. Those with wheat allergies miss out on major fiber sources, like whole grains and bran [90]. Many of these fibers are considered “prebiotic” and promote the growth of health gut bacteria [90]. Not surprisingly, it has been seen that the loss in fiber significantly reduces the abundance of fiber-degrading bacteria, resulting in a reduction in SCFA levels [91]. Luckily, within resent years, there has been in increase in wheat free, or gluten free, products in the food industry [92]. While these products have been made for gluten intolerance in mind, those with an IgE-mediated wheat allergy or gluten sensitivity may also benefit. There are currently no data on the interactions of the microbiome with wheat allergies. This may be of great interest, due to the large fiber components of many wheat products.

### 5.7. Soy

Soy is a complete protein, with sensitization normally occurring from the soybean hull proteins (Table 1) [25]. A total of 0.4% of children in the U.S. have a soy allergy, and 50% of those tend to outgrow it by adulthood [93]. Interestingly, one report found that eighty-eight percent of those with soy allergies also have peanut allergies [93]. Soy lecithin is a very common additive included in many food items. For instance, it is used as an emulsifier in chocolate and dairy products and filler in baked goods to reduce fat content. Unfortunately, not much work has been completed on the effect of avoiding soy consumption on the gut microbiome, nor has there been any research on the potential role of the gut microbiome in soy allergy. 

### 5.8. Finfish

The consumption of fish is beneficial, as fish are high in omega-3 and omega-6 fatty acids. Omega-3 is well known to have many health benefits and has been seen to help with other IgE-mediated conditions [94]. Fish oil tablets may be taken, but many of these capsules are heavily oxidized before reaching the consumer. Finfish allergens, as with shellfish, may not appear until adulthood. However, this may be due to a lack of fish in childhood diets. The main proteins that cause allergy are parvalbumins, with different variations found as the main protein in different fish (Table 1) [24]. Interestingly, previous studies have found that antacids encourage sensitization to parvalbumin in mouse models [95]. This is likely due to the increasing the pH in the stomach, allowing the intact protein to move along into the gut. As with wheat and soy, there is no previous research on the interactions of finish allergy and the microbiome.

### 5.9. Common or Unique—Summary of Work

Shown below in Table 2 is a summary of the work presented in this review. There are currently no studies of the role or interactions of the gut microbiome with wheat, soy, or fish allergies. It is possible that some of the microbiome changes and allergy mechanisms may overlap between these allergies and those described above. It is not uncommon for individuals to have multiple food allergies. It is also interesting to note the possible change multiple allergies may have on microbiome dysbiosis vs. a singular allergy; however, this has not been investigated. Therapies and supplements (Table 2) may be utilized as functional food additives.

Many of the same mechanisms were proposed for each of The Big Eight, with some contradicting each other (Table 3). Many of the theories revolve around Clostridia and its abilities to regulate T cells. Due to its importance as the most prevalent childhood allergen, cow milk has been the most studied food allergen in term of microbiome association. Mechanistic differences between other allergens may occur, as currently there are not enough data to defer the theory. Importantly, there are currently no data available showing the effect that the gut microbiome has on wheat, soy, and finfish allergies.

## 6. There Is More Than Meets the Gut: Gaps and Future Perspectives

### 6.1. Viral and Fungal Interactions

While not given the same amount of attention as the bacterial world in the gut, viruses and fungi may assist in the development and/or treatment of food allergy. Asthma, hay fever, and peanut allergy were found inversely related to hepatitis A, herpes simplex virus 1, and *Toxoplasma gondii* infections [97]. Previous research has shown murine norovirus may drive allergic disease, using egg ovalbumin, through changes in normal dendritic cell function [98]. Interestingly, Kernbauer et al. found murine norovirus infection of germ-free mice can replace the beneficial effects of commensal bacteria [99]. Infection of murine norovirus 4 in non-obese diabetic mice was reported to lead to positive changes in mucosal immunity, altering Tuft cell makers, cytokine secretion and mucosal antibodies [100]. However, others have opposite conclusions, reporting that norovirus and rotavirus infection may lead to increased protein absorption and sensitization in the gut [101]. Diets of varying fiber and fat have been shown to change the virome. However, it is not well understood how these changes affect the human body [102]. While dietary fungi have been known to cause allergic reactions, commensal fungi have not been studied for a possible cause or adjunct of disease. Allergy research must be conducted on these missing areas, as major parts of the human microbiome include viral and fungal organisms.

### 6.2. Clostridiales Cocktail

Next-generation probiotics is a term used to describe the use of commensal bacteria, rather than lactic acid food associated bacteria, as probiotics [103]. There is a clear trend with the involvement of the Firmicutes phylum, and more so with the Clostridiales order, involved in the sensitization and treatment of food allergies. Clostridiales use as a therapy and a next-generation probiotic should be further investigated. Throughout this review, Clostridiales has been mentioned several times in multiple food allergens to be a beneficial bacteria. A strain-specific difference in beneficial effects in *Roseburia* and *Blautia* give further evidence to the theory that not all Firmicutes, or SCFA-producing bacteria, have the same effect on the body [42]. *Dorea* is discussed through multiple studies for possible beneficial effects. For example, one study characterized 17 different Clostridia strains to have positive health effects on food allergy [64]. Further investigation of the effects of Clostridiales on food allergies and use as a treatment option would be valuable, especially regarding specific species, dosage, and timing of administration.

### 6.3. Exparimental Design and Analysis

Most microbiome studies utilize the 16S ribosomal RNA (rRNA) sequencing technique. However, only certain levels of taxonomy can be reached, with many only going down to the genus level. Virus and fungal communities are completely missed. As stated above, species- and strain-level differences likely contribute a larger part of allergy disease than previously discovered. Rarely functional characteristics of bacteria are further explored as well, with many reports simply stating the taxonomical results from sequencing. With most gut microbiome studies, and those discussed in this review, only the large intestinal microbiome is analyzed, as stool is used for sampling. However, with food being digested in the small intestinal, it is important to observe microbial and metabolic changes in this region as well. These important research details are required to further understand the complexly of the immune system and the entire microbiome.

## 7. Conclusions

Food allergies have an enormous impact on quality of life. The rates of those with food allergies have notably increased in the past decade, suggesting that environmental factors are driving most of the increase [1]. Numerous animal and human studies have supported this theory with the connection of the gut microbiome. Alteration of the microbiota across The Big Eight allergies shows a consistent unique signature involving the Firmicutes phylum. Interaction of the microbiome and microbial metabolites (SCFAs) with host immune response is likely the mechanism by which the microbiome affects food allergy. The addition of probiotics, both traditional (*Lactobacillus* and *Bifidobacterium*) and next generation (Clostridia), along with other dietary interventions have shown significance in helping prevent and treat disease. This knowledge may be used to develop products utilizing these as functional additives. Further research must be conducted to identify potential differences in mechanisms, prevention, and treatment between different antigens.

## Figures and Tables

**Table 1:** Proteins present in The Big Eight foods known to induce allergy.

Cow milk	Casein, whey, bovine serum albumin, immunoglobulin G heavy chain and a-lactalbumin [19]
Hen’s egg	Whites: Ovomucoid, ovalbumin, ovotransferring, and lysosome [20] Yolk: Livetin, vitellenin and apoprotein B [20]
Peanut	Cupin(Ara h 1, 3), prolamin(Ara h 2, 6, 7, 9), profilin(Ara h 5), bet v-1-related proteins(Ara h 8), oleosin(Ara h 10,11) and defensin(Ara h 12, 13) [21]
Tree nut	Vicilin, 2S albumin, nsLTP, legumin, bet v 1-like, oleosin, 60 s acidic ribosomal prot. P2, manganese superoxide dismutase and profiling [22]
Shellfish	Tropomyosin, arginine kinase, myosin light chain, and sarcoplasmic calcium-binding protein [23]
Finfish	Parvalbumins, enolase, aldolase, tropomyosin, vitellogenin [24]
Soy	Hull proteins, kunitz trypsin inhibitor, glycinin, α-subunit of β-con-glycinin, and 50-kd protein with homology to chlorophyll A-B binding protein and starvation-associated message [25]
Wheat	α-Amylase/trypsin inhibitor, album, globulins, serine proteinase inhibitor, lipid protein transfer, thioredoxin, peroxidase, gliadin, thiol reductase, and thaumatin-like protein [26]

**Table 2:** Summary of trends thought to promote/reduce risk or treat allergies for each of The Big Eight.

	Promote Risk	Reduce Risk	Therapies and Supplements
Cow milk	^d^ General antibiotic use before and during pregnancy ^h^ [38]	^a^ Increase in diversity ^h^ [39]	^a,b^*Bifidobacterium infantis* CGMCC313-2, 5 × 10^10^ CFU/mL for 6 days ^m^ [53]
	^d^ Short-chain fatty acid ^m^ [3]	^a^ Breastfeeding ^h^ [32]	^c^*Lactobacillus rhamnosus* GG, 4.5 × 10^7^–8.5 × 10^7^ CFU/g of formula for 6 months ^h^ [42]
	^e^*Clostridium coccoides*^h^ [45]	^a,e^ Firmicutes ^h,m^ [39,42,43]	^c^*Lactobacillus rhamnosus* GG, 1.4 × 10^7^ CFU/100 mL of formula for 6–12 months [47]
	^e^*Atopobium*^h^ [45]	^a,e^ Clostridia ^h,m^ [39,42,43]	
	^e^*Lachnospiraceae*^h^ [42]	^e^*Oscillospira*^h^ [42]	^a^*Bifidobacterium breve* M-16V, 2 × 10^9^ colony forming unit (CFU)/g for 7 weeks ^m^ [52]
	^e^*Ruminococcaceae*^h^ [42]	^e^*Roseburia*^h^ [42]	
	^e^*Lactobacilli*^h^ [4]	^e^*Blautia*^h^ [42]	
	^a^*Anaerostipes caccae*^h,m^ [43]	*^a,^*^e^*Bacteroides*^h,m^ [36]	
	^a,e^*Bifidobacterium*^h,m^ [28,36]	^e^*Enterobacteria*^h^ [4]	
Hen’s egg	^e^ Increase in diversity ^h^ [59]	^e^*Leuconostocaceae*^h^ [59]	^a^*Bifidobacterium longum*, 5 × 10^9^ CFU daily for 36 days ^m^ [62]
	^e^*Lachnospiraceae*^h^ [6,59]		^a^ Clostridiales consortium, 5 × 10^7^ CFU twice weekly for five weeks ^m^ [63]
	^e^*Streptococcaceae*^h^ [59]		^a^*Subdoligranulum variabile*, 2.4 × 10^6^ CFU twice weekly for five weeks ^m^ [63]
	^e^*Lactobacillaceae*^m^ [6]		^a^ Bacteroidales consortium 5 × 10^7^ CFU twice weekly for five weeks ^m^ [63]
	^e^*Rikenellaceae*^m^ [6]		^a^*Bifidobacterium bifidum,* 0.2% lyophilized for 8 weeks ^m^ [61]
	^e^*Porphyromonadaceae*^m^ [6]		^a^*Lactobacillus casei,* lyophilized for 8 weeks ^m^ [61]
	^d^*Proteobacteria consortium*^m^ [63]		^a^*Escherichia coli* lyophilized for 8 weeks ^m^ [61]
	^e^*Ruminococcaceae*^h^ [59]		^a^ Clostridia via monocolonization ^m^ [64]
Peanut			^c^*Lactobacillus rhamnosus* and peanut oral immunotherapy, 2 × 10^10^ CFU once daily with peanut protein for 18 months ^h^ [71]
			^c^*Lactobacillus rhamnosus* GG and peanut oral immunotherapy, 2 × 10^10^ CFU of *L rhamnosus* CGMCC 1.3724 and 2 g of peanut protein once daily for 18 months ^h^ [72]
			^a^ High-fiber diet and vitamin A for 2 weeks ^m^ [12]
			^a^ Direct feeding of acetate and butyrate for 3 weeks ^m^ [12]
			^a^ Clostridia via 2 oral gavages, once a week ^m^ [75]
Tree nuts	^e^ Bacteroidales ^h^ [80]	^e^ Clostridiales ^h^ [80]	
	^e^*Bacteroides fragilis*^h^ [80]	^e^*Prevotella*^h^ [80]	
	^e^ Bacteroidales ^h^ [80]	^e^*Ruminococcaceae*^h^ [80]	
Shellfish	^e^*Ralstonia*^m^ [87]	^e^*Dorea*^m^ [87]	^a,b^*Bifidobacterium infantis*, 10^7^ CFU/mL for 20 days ^m^ [87]
			^b^*Bifidobacterium lactis*, 10 mL (1 × 10^6^/mL) daily for 3 months ^h^ [88]
			^a^*Bifidobacterium longum,* 2 × 10^10^ CFU for 22 days ^m^ [89]
			^a^*Bacillus coagulans* 2 × 10^10^ CFU for 22 days ^m^ [89]
Finfish			
Wheat	No data present		
Soy			

^a^ Prevention of sensitization. ^b^ Therapeutic during allergic reaction. ^c^ Increased oral tolerance. ^d^ Worsened Sensitization. ^e^ Bacteria found in the gut of either FA or health individuals. Mouse study ^m^. Human study ^h^. Colony forming unit (CFU).

**Table 3:** Summary of proposed mechanistic actions of the effect that the gut microbiome has on food allergies.

Proposed Mechanisms	Allergens Involved
Increase in diversity strengthens and stablishes immune system. ^h^ [39]	Cow milk
Exclusive breastfeeding prevents the introduction of cow milk required for sensitization. ^h^ [32]	Cow milk
Firmicutes, those in the Clostridia order and those which increased butyrate production, which regulate colonic regulatory T cells. ^mh^ [12,39,42,43,63,64]	Cow milk, Hen’s egg
Those in the Clostridia order regulate innate lymphoid cell function to alter epithelial permeability and reduce allergen uptake into the systemic circulation. ^m^ [75]	Cow milk
Pyruvate metabolism from those in the Clostridia order and *Leuconostocaceae* family depletes uric acid, which inhibits the activity of xanthine oxidase. ^mh^ [43,59,75]	Cow milk, Hen’s egg
*Bifidobacterium* and *Bacteroides* invoke *foxp3* gene activation, which is responsible for the development and function of regulatory T cells. ^mh^ [28,36,52,53]	Cow milk
*B. infantis* increased the abundance of butyrate-producing bacteria, which in turn suppress the inflammatory responses triggered by Th2 cytokines. ^m^ [53,96]	Shellfish
Lactobacillus rhamnosus GG promotes growth of short-chain fatty acid-producing bacteria. ^h^ [42]	Cow milk, Peanut
*Bifidobacterium longum* induces apoptosis to bonded mast cells. ^m^ [62]	Hen’s egg
Antibiotics decrease level of beneficial bacteria. ^h^ [38]	Cow milk
Firmicutes and those in the Clostridia order increased butyrate production, which in turn increases the permeability of the intestinal mucosa. ^mh^ [28,44]	Cow milk
*Bifidobacterium longum and Bacillus coagulans* regulate gut arginine metabolism pathways and the metabolites of aspartate and arginine may be critical for prevention of food allergy. ^m^ [89]	Shellfish

Mouse study ^m^. Human study ^h^.
